# Profound Weakness and Blurry Vision in a Pandemic: A Case Report

**DOI:** 10.5811/cpcem.2021.3.51104

**Published:** 2021-05-06

**Authors:** Jacy M. O’Keefe, Kristi J.H. Grall

**Affiliations:** * HealthPartners/Regions Hospital, Department of Emergency Medicine, St. Paul, Minnesota; † University of Minnesota, Department of Emergency Medicine, Minneapolis, Minnesota

**Keywords:** Emergency medicine, case report, neuro-Behçet’s disease, SARS-CoV2

## Abstract

**Introduction:**

Neuro-Behçet’s disease (NBD) is a manifestation of Behçet’s disease, a relapsing inflammatory multisystem disease. Data on patients with autoimmune disease in the setting of severe acute respiratory syndrome coronavirus-2 (SARS-CoV-2) is limited.

**Case Report:**

We discuss the case of a 22-year-old male with SARS-CoV-2 who presented to the emergency department with weakness and vision changes. Brain imaging showed enhancing lesions. History revealed possible autoimmune disease. A diagnosis of NBD exacerbated by SARS-CoV-2 was made.

**Conclusion:**

Patients with SARS-CoV-2 are presenting with exacerbations of systemic illnesses. Although NBD is uncommon, medical professionals need to consider this in the differential of central nervous system disorders, as it is a potentially treatable condition.

## INTRODUCTION

Neuro-Behçet’s disease (NBD) is one of the more serious manifestations of Behçet’s disease (BD), a relapsing inflammatory multisystem disease.^1^ Data on patients with systemic autoimmune disease in the setting of severe acute respiratory syndrome coronavirus-2 (SARS-CoV-2) is limited, but it has been hypothesized that those with underlying disease processes may have worse outcomes.^5^ However, little has been published on this topic.

## CASE REPORT

A 22-year-old Black male with a past medical history of asthma and migraines presented to the emergency department (ED) with diffuse weakness and vision changes. Paramedics reported that they were called to the patient’s home by his mother due to profound weakness. The patient’s history was limited by his somnolence, but he noted progressive weakness, intermittent headaches, and blurred vision in his left eye over the prior three weeks. The patient denied drug or alcohol use or recent head trauma, and reported his immunizations were up to date. He denied a fever, neck pain or stiffness, recent sick contacts, urinary incontinence, saddle anesthesia, or numbness/paresthesia. His mother also reported significant anorexia and weight loss. She stated he had been living in a home with possible black mold. The patient delayed seeking medical attention due to the current SARS-CoV-2 pandemic but agreed to be evaluated after he lost the ability to ambulate.

Vital signs were as follows: blood pressure of 111/75 millimeters of mercury; heart rate of 74 beats per minute; temperature of 97.5°F, respiratory rate of 10 breaths per minute; and oxygen saturation of 99%. Physical examination was notable for left ocular strabismus and anisocoria, with the left pupil measuring 3 millimeters (mm) and the right pupil measuring 5 mm, both reactive to light. He had normal extraocular movements. The patient was symmetrically weak with notable bradykinesia; otherwise the neurologic exam was without abnormality. The remainder of the physical exam was noted to have no significant external findings of head trauma, conjunctivae were clear, Brudzinski’s sign was not present, and there was no nuchal rigidity or photosensitivity. He had no rashes on his exposed skin.

Initial laboratory analysis including a complete blood count, basic metabolic panel, lactate, magnesium, thyroid stimulating hormone, ethanol level, and urinalysis was without abnormality. A chest radiograph and non-contrast computed tomography (CT) of the head showed no abnormalities. Polymerase chain reaction was positive for SARS-CoV-2.

Due to the patient’s profound weakness and abnormal ocular findings raising concerns for possible underlying demyelinating disease, malignancy, stroke, or infection, the decision was made by the emergency physician to obtain a brain magnetic resonance imaging (MRI) (Image). With the exception of worsening drowsiness, the patient’s clinical status was unchanged during his ED course.

Upon further questioning, the patient reported recurrent oral and genital ulcers, headaches, and vision problems. He denied any respiratory symptoms and his vital signs remained stable in the setting of SARS-CoV-2 infection. He was subsequently admitted to the hospital.

The differential diagnosis included neurosyphilis or other sexually transmitted infection, human immunodeficiency virus, central nervous system (CNS) lymphoma, sarcoidosis, lupus, other autoimmune disease, infectious etiology (toxoplasmosis, Lyme disease, Whipple disease, tuberculosis, neurocysticercosis, cryptococcus, blastomycosis, histoplasmosis, abscess, and SARS-CoV-2), and paraneoplastic process. Per the radiology report, the lesions did not appear to be consistent with multiple sclerosis.

**CPC-EM Capsule**

What do we already know about this clinical entity?

*Data is limited on autoimmune disorders, such as Behçet’s disease (BD), in the setting of severe acute respiratory syndrome coronavirus-2 (SARS-CoV-2).*

What makes this presentation of disease reportable?

*Neuro-Behçet’s disease (NBD), a rare manifestation of BD, in a patient with underlying SARS-CoV-2 is an unusual diagnosis.*

What is the major learning point?

*Neuro-Behçet’s disease is a rare but treatable manifestation of BD that may be exacerbated due to delay in seeking medical care during the coronavirus disease 2019 pandemic.*

How might this improve emergency medicine practice?

*Early diagnosis of NBD can result in improved patient outcomes.*

During the patient’s admission to the hospital, consultations with rheumatology, infectious disease, and neurology were obtained. Additional history obtained during his admission included a report that he had been previously worked up for Crohn’s disease, but no formal diagnosis had been made. Additionally, the patient had previously been diagnosed with uveitis. His mother denied any family history of autoimmune or neurologic disorders. A CT of the chest, abdomen, and pelvis was performed, which did not reveal malignancy or abnormality. A CT cerebral venography was negative for thrombosis. A testicular ultrasound was normal. A lumbar puncture was performed and cerebrospinal fluid (CSF) analysis was significant for neutrophilic leukocytosis with elevated protein, but was otherwise negative for viral, bacterial, fungal, parasitic, or malignant etiologies. Neurosurgery did not recommend biopsy of the lesions due to the location.

After ruling out other etiologies, a diagnosis of BD and NBD was made, using the International Clinical Criteria for Beh**ç**et’s Disease (ICCBD) (the condition is clinically diagnosed rather than serologically) and the International Consensus Recommendation (ICR) criteria for NBD diagnosis, respectively (Table). Per the ICCBD, the patient must have recurrent oral ulcerations as well as two of the following: recurrent genital ulcerations; eye lesions; or skin lesion to make the diagnosis of BD. The patient received points for recurrent oral and genital ulcerations and uveitis.^1^ According to the ICR, he met criteria for NBD due to neuroimaging findings, CSF analysis, and the lack of a better explanation for the neurological symptoms.

## DISCUSSION

Neuro-Beh**ç**et’s disease is a serious manifestation of BD, which is a relapsing inflammatory multisystem disease.^1^ Between 5–30% of BD patients will present with neurological changes on initial evaluation and up to 49% will develop neurological involvement at some point in their disease course.^7^ Although serious neurologic flares are considered rare, the manifestations of NBD can be severe with a high mortality rate. This condition is potentially treatable; so medical professionals need to consider NBD in the differential diagnosis of inflammatory, infective, or demyelinating CNS disorders. When patient’s present with neurologic changes and a reported history of recurrent oral or genital ulcers, uveitis, or other systemic features of BD, NBD should be considered.

Neuro-Beh**ç**et’s disease is often compared with multiple sclerosis (MS); accordingly, MS was high on the differential for this patient. Certain neurological features such as sensory complications, optic neuritis, internuclear ophthalmoplegia, limb ataxia, and cerebellar dysarthria are more common in MS, while headaches, motor symptoms, pseudobulbar speech, and cognitive-behavioral changes are more common in NBD.^2^ Unmatched CSF oligoclonal bands are present in the majority of MS patients and are uncommon in NBD.^4^ In NBD, CSF will show more white blood cells with neutrophils predominating, while in MS neutrophils are scarce and lymphocytes generally predominate.^3^ Radiologically, NBD usually involves the white matter, brainstem, basal ganglia, and thalamus, whereas in MS the lesions are periventricular, with infrequent involvement of the basal ganglia or internal capsule.^8^ Cerebral MRI findings often reveal T1 iso/hypointense and T2 hyperintense lesions in NBD, similar to MS.^8^

There is no level I evidence on treatments for NBD and, therefore, no US Food and Drug Administration- approved medications. For an acute or sub-acute parenchymal NBD flare, high-dose intravenous (IV) corticosteroids (one gram methylprednisolone) is recommended for 4–5 days, followed by an oral corticosteroid for up to six months (60 milligrams [mg] prednisone daily). Colchicine, azathioprine, tumor necrosis factor (TNF)-alpha inhibitors (adalimumab, infliximab, among others), or interferon alpha can be considered. These medications can improve or completely resolve lesions of NBD if initiated early enough in the disease course.^1^

Data on patients with systemic autoimmune disease and concomitant SARS-CoV-2 is limited, but reports have recently been released in Europe.^5^,^6^ In one case series, 10 SARS-CoV-2 positive BD patients were evaluated.^4^ Except one patient who was off treatment , all of the other nine were using one of the following drugs either alone or in combination: colchicine (n=5); azathioprine (n=3); anti-TNF agents (n=3); or prednisolone (n=2). Of the patients involved in the case series, in addition to skin mucosa lesions, four had eye involvement, one had both eye and neurological involvement, and one had large vessel disease. The patient in this series who was off treatment ultimately died secondary to respiratory failure. Another patient had NBD and was hospitalized with deep venous thrombosis. Four patients were diagnosed with SARS-CoV-2 pneumonia. Three of these were hospitalized with one requiring intensive care unit admission. Three patients had exacerbations of oral ulcers or arthralgias. Four patients had no complications associated with BD during their course with SARS-CoV-2.

During the admission of our patient, he continued to be weak and developed aphasia, right hemiparesis with spasticity, uncontrollable laughter, and urinary incontinence. Initially, steroids were avoided due to SARS-CoV-2, but with worsening neurologic symptoms, it was decided that the benefits outweighed the risks and he was started on one gram IV methylprednisolone daily for five days. He was also started on anticoagulation therapy due to both BD and SARS-CoV-2 being associated with hypercoagulable states. Prior to discharge after a 16-day admission, he had improvement in his neurologic symptoms, and repeat MRI demonstrated significant improvement with near resolution of brain lesions. He was discharged on 40 mg subcutaneous adalimumab every two weeks and 60 mg oral prednisone daily with close rheumatology follow-up. Although the patient never developed typical symptoms of SARS-CoV-2, he did have a severe exacerbation of NBD, which may have been in part due to this underlying viral illness and his delay in seeking medical attention due to the pandemic.

## CONCLUSION

More research is needed to understand the relationship between SARS-CoV-2 and the effect it has on autoimmune diseases, such as Behçet’s disease. It must also be noted that during the COVID-19 pandemic patients were initially avoiding seeking medical care, resulting in critical presentations requiring emergent intervention.

## Figures and Tables

**Image:**
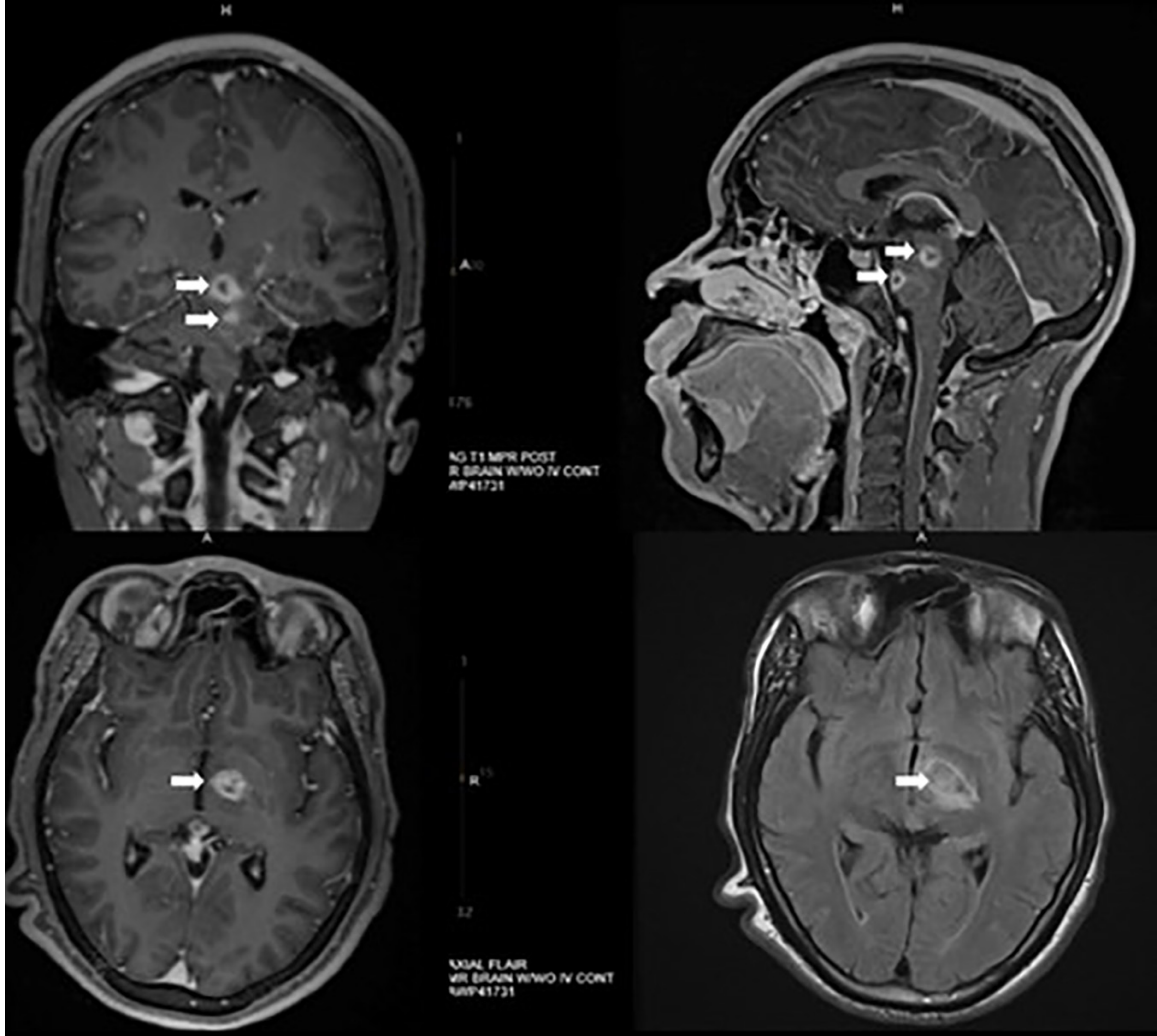
Magnetic resonance imaging of the brain with/without intravenous contrast: multifocal abnormal enhancing lesions (white arrows) in the bilateral cerebral hemispheres, left thalamus, and brainstem.

**Table:** International Consensus Recommendation criteria for neuro-Behçet’s disease.^1^

The ICCBD states patients must present with: Recurrent oral ulcerations (apthous or herpetiform) at least three times in one year. Additionally, patients must present with any two of the following: Recurrent genital ulcerations Eye lesions (uveitis or retinal vasculitis) observed by an ophthalmologist Skin lesions Positive pathergy test read by a physician within 24–48 hours The ICR for NBD states definite NBD meeting all of the following three criteria: Satisfy the ICCBD Neurological syndrome (with objective neurological signs) recognized to be caused by Behçet’s disease and supported by relevant and characteristic abnormalities seen on either or both: Neuroimaging CSF No better explanation for the neurological findings Probable NBD meeting one of the following two criteria in the absence of a better explanation for the neurological findings: Neurological syndrome as in definite NBD, with systemic BD features but not satisfying the ICCBD A non-characteristic neurological syndrome occurring in the context of ICCBD-supported BD

*NBD,* neuro-Behçet’s disease; *BD,* Behçet’s disease *CSF,* cerebrospinal fluid;* ICCBD,* International Clinical Criteria for Behçet’s Disease; *ICR,* International Consensus Recommendation.
